# Root Infection and Systemic Disease

**Published:** 1918-11

**Authors:** 


### ROOT INFECTION AND SYSTEMIC DISEASE

These extracts are taken from the October *Dental Items of Interest,* which prints a series of replies as to the relations of systemic disease and tooth root infections.

Dr. Julio Endelman, editor of the *Pacific Gazette,* says: I am satisfied that oral focal infections are sometimes responsible for systemic involvements, but I am not convinced that every focus of infection—intraosseous or extraosseous infection—is liable to result in lesions of organs or tissues of the body removed from the original seat of infection. My evidence is in the shape of clinical results. I have in the last two years treated dozens of patients with incipient tubercular lesions, or with reactivated tubercular lesions, who have responded most beautifully to the treatment to which I have subjected them.

The fact that the micro-organism is found in an organic lesion, or muscular lesion, or joint lesion, or heart lesion, or kidney lesion, and that the same type of organism is found in the focus of infection in the mouth is no convincing proof to me that the original oral lesion is the source of the systemic involvement. The mere finding of the organism does not establish convincingly, in my conception, the relation between the focal infection and the systemic involvement.

The editor of *Items of Interest* submitted two questions to a number of experts and requested them to give brief replies. These replies are very interesting and instructive and we quote them quite completely. The questions were as follows:

"First, do you believe that such diseases as arthritis, heart disease, etc., can be caused by infections at the ends of tooth roots? If so, will you state just what you consider the absolute proof of this theory?"

"Second, do you think that arthritis or heart disease can be caused from pus discharging from pockets around teeth or from fistulae which lead to apex infections? If so, will you state definitely the eidence upon which you base this belief?"

Dr. F. B. Moorehead: It would hardly seem necessary at this time to debate the question of focal infection. Medical literature abounds in interesting case reports and clinical observations made by the best men in medicine illustrating the relationship between certain focal infections and more or less generalized chronic infections. One may find in the literature numerous illustrative cases of appendicitis, endocarditis, etc., following tonsilitis. Billings, in his monograph, "*Focal Infections,*" reviews the development of the increasing appreciation of the role of focal infections and their influences dating from the days of Schemmelweiss.

In chronic infections including the groups involving joints, muscles, nerves, heart, etc., the bacterial cause is well known. Post-mortem findings in ulcerative endocarditis reveals the type of streptococcus always associated with that lesion found both in the heart valves and in the blood stream. The aspiration of chronic joints shows the streptococcus. Removal of pieces of tissue from joints or contiguous lymph nodes reveals the streptococcus. The literature of the past five years demonstrates eloquently the fact that the streptococcus is the organism producing these several forms of chronic infection. The literature of the past five years also demonstrates that the streptococcus is intimately associated with chronic lesions of the tonsil, jaws, accessory nasal sinuses, etc.

There is no more definite means of demonstrating the cause and effect of infection than through the process of Koch's postulates. Rosenow has made such a complete demonstration of the production of chronic infections in animals from organisms obtained from the human, recovering the organism again from the lesions in the animal, that there scarcely seems to be any room open for argument.

The fact that one may take a culture from a tonsil or chronic alveolar abscess in a patient suffering from general arthritis and inoculate a guinea pig or rabbit or dog and produce an arthritis in that animal is an unanswerable argument. While on the other hand, the fact that one may take a similar culture and not produce an arthritis does not necessarily prove anything. If every human being should succumb to every opportunity for infection, the human family would be decimated in a very short period of time. One may find the typhoid baccillus, the diphtheria bacillus, tubercle-bacillus or the pneumococcus, in the secretions of the mouth or gastro-intestinal tract in an individual enjoying perfect health.

Certain well-defined conditions must be present before harm is done by the invasion of any organism. Much depends on, first, the virulence of the organism, and second, the susceptibility of the individual. Immunity is relative, not absolute, and varies from day to day, or even from hour to hour under certain conditions.

The details upon which this whole question is adjudicated are elemental:

I. In the vast majority of patients suffering from chronic infections, one finds a local focus somewhere in the body. This may be in the tonsil, about the joints, nose and accessory sinuses, appendix, gall bladder, gastro-intestinal tract, etc.

II. Marked improvement follows removal of these foci in a large percentage of cases, the improvement relating very definitely to the chronicity of the case. It is perfectly obvious that in a patient suffering for ten years from a chronic arthritis, the removal of the original focus may not necessarily modify the condition because of the fact that secondary depots of infection have been established in contiguous lymph nodes, in the joints and elsewhere, and also because of the fact that certain tissue changes once developed are likely to persist.

III. The production of heart lesions, lesions of the appendix, joints, muscles, nerves, etc., in animals through inoculation with organisms taken from chronic tonsils, alveolar abscess, etc., is well illustrated in the brilliant work of Rosenow, and verified by other well-trained technicians.

The old theory of auto-infection is no longer tenable. In order to do harm invading organisms must enter the circulating fluids of the body through a breech in either the integument or mucous membrane. Pus, therefore, discharging from a chronic alveolar abscess and entering the stomach and intestines will not produce a lesion unless the organism in the pus finds a broken surface somewhere in the gastro-intestinal tract, and even then will probably not produce any lesions because of the fact that in order to be effective the organism must find a focus where it can multiply over a longer or shorter period of time. Only the organism producing acute infection will probably do harm by being admitted through a break in the integument or mucosa.

Dr. Thos. P. Hinman: I do believe that such diseases as arthritis, heart disease, etc., are caused by infection at the ends of the roots of the teeth. My reason for such belief is that the work of Hartzell, Rosenow and many other investigators have proved that the injection in animals of bacteria that came from cultures made from the ends of the roots of teeth or the sacs around the ends of the roots of teeth, has produced similar diseases in these animals. Then, too, in my private practice I have cleared up these apical conditions by various methods and have had arthritis and various other diseases that are caused by this infection, to clear up completely without the patient's receiving any other treatment.

I believe that arthritis and heart disease can be caused from discharge from pockets around the teeth and possibly from fistulae which lead to the apical infections. It is true, in such discharge, we have a mixed infection of staphilococcus and streptococcus, but if the tissues in the deep pockets in pyorrhea are removed, the same character of tissue is found that we find around the apices of certain teeth, where there is only one infection and that streptococcus.

I am not so certain about heart infections, but I have seen a number of cases of arthritis clear up after the pyorrhea was completely eradicated from the mouth. My experience and observation have been that the so-called granuloma around the apex of the teeth which has no external opening is the more dangerous of the two infections.

Dr. Thos. B. Hartzell: It seems to me that my published utterances on the questions you have asked me furnishes all the evidence you need in answer to them.

An examination of the published evidence from many other pens, save mine, amply confirms the fact that arthritis and heart disease are caused by infections from bacteria, either from tooth-root ends or pyorrhea pockets.

These statements have been verified by culturing from the diseased human organs and obtaining streptococcus viridans from them. These germs in turn have produced animal lesions caused by the introduction of streptococcus viridans into the blood streams of these animals. The pathologists concede these statements to be true. As far as I am personally concerned, I am thoroughly convinced beyond any shadow of doubt that heart disease is a streptococcal lesion. I believe it because I have found the streptococcus many times in diseased human hearts, and have grown it from the heart and from the dental abscess on the same culture media. I am absolutely certain, in my own mind, that neither of the cultures from the dental abscess or from the heart were contaminated, as we had control media, which remained sterile for every one of these experiments.

Col. Frank Billings, Medical Corps, U. S. A.: I am one member of the medical profession who believes that systemic disease may be caused by a focal infection, whether it be related to the roots of the teeth or to any other part of the body where the infection is confined and undrained. I have had this idea for many years—twenty-five or more—and as you may possibly know have made, with the co-operation of my colleagues and students, investigations upon both man and animals. This work, including investigations, have been published in many articles and especially collected in a book entitled "*Focal Infection,*" published by D. Appleton & Company in 1916, with second edition in 1917.

Therefore, in answer to your questions, I would say:

1. I believe that systemic disease may occur through confined infection at the ends of the roots of the teeth.

2. When the infection about the tooth is drained into the mouth or elsewhere through a sinus, or when the exudate caused by the infection escapes through a sinus or down the side of the root of a tooth it usually will not cause systemic disease. The pus and other elements in the exudate which so escapes into the mouth may cause local disturbance of the mucous membrane of the mouth, or if swallowed may cause some disturbance of the stomach, but the action of the gastric juice will, as a rule, protect the individual against systemic infection by the chemical action of the gastric juice upon the exudate and the contained bacteria.

3. Systemic infection from any source, focal or other, is naturally related to resistance of the individual; that is, of the host. Many individuals are so strong, healthy and resistant that the bacteria of the focal infection, even though they reach the blood stream, are overcome by the natural resisting powers or the defenses of the body. This principle applies to young people and to vigorous adults. Anything which reduces the defenses of the host, whether young or old, such as undue exposure to cold, fatigue, unhygienic surroundings, or any other debilitating influence may break down the resistance and the focal infection may then be operative.

Major Harlow Bowles, Medical Corps, U. S. A.: I do believe that general infections may be inaugurated from infected foci located at the ends of the teeth. My reasons are that I have seen several instances where symptoms and signs of these infections were entirely relieved by the elimination of the primary focus. I believe that this is also substantiated by analogous pathological processes, as in infected tonsils, undrained abscesses, etc. I believe that the present tendency is, however, to overestimate the frequency with which this occurs.

Second, from my knowledge of similar processes with which I am familiar, I think it very unlikely that generalized infection should follow in such an instance as you describe. Of course, it is theoretically possible, but very unlikely, in my opinion.

In regard to the extraction of teeth, I would answer that I do not feel that so radical a measure is justified unless actual disease with symptoms are present, nor until all other possibilities have been considered and eliminated.

Wm. H. Park, M. D., Department of Health, New York City: I can only answer your questions in a very general way. I have not begun to have had enough experience to feel justified in having any strong beliefs. There seems to me no question that general infections follow from foci of infection in or about the teeth. Personally, I have some doubt as to whether organisms that are discharged into the mouth and swallowed, do no harm after reaching the stomach. I confess, however, that this is a belief and not founded on any definite facts. In a general way, I think the teeth that have a discharging sinus are safer than an equal infection which is closed. I do not believe anyone knows what proportion of teeth gives trouble; probably a very small proportion. This lack of definite knowledge makes it extremely difficult to decide whether useful teeth should be removed except in any person having infections which are known to come from infected teeth. In any individual, the focus may have been in the tonsils or other tissues of the body, as well as in the suspected tooth.

This very general answer is the best I can give your questions.

